# Synergistic Application of Cytidine Monophosphate and Sodium Chloride for Enhanced Co-Production of Astaxanthin and Fatty Acids in *Haematococcus lacustris* Motile Cells Under High-Light Stress

**DOI:** 10.3390/md24080285

**Published:** 2026-08-19

**Authors:** Xiaoyuan Su, Hailiang Xing, Kai Liu, Ya Zhao, Lijin Dong, Ziyan Zhou, Na Zhou, Xue Sun, Liuquan Zhang, Nianjun Xu, Chaoyang Hu

**Affiliations:** Key Laboratory of Aquacultural Biotechnology of Ministry of Education, School of Marine Sciences, Ningbo University, Ningbo 315832, China

**Keywords:** secondary cell wall, microalgal biorefinery, metabolic reprogramming, transcriptomics, gas chromatography–mass spectrometry

## Abstract

This study evaluated the synergistic effects of sodium chloride (NaCl) and cytidine monophosphate (CMP) on enhancing the co-production of astaxanthin and fatty acids while suppressing secondary cell wall (SCW) formation in *Haematococcus lacustris* (synonym: *H. pluvialis*) under high-light stress. An orthogonal design identified the optimal combination (0.5 g/L NaCl and 0.5 mM CMP), which significantly increased astaxanthin yield by over 35.6% and total fatty acid yield by 28%, while maintaining 96.8% of cells in motile state (SCW-deficient). Physiological analyses revealed elevated reactive oxygen species levels, concomitant with higher actual photochemical efficiency (Fv′/Fm′) and relative electron transport rates II (rETR(II)) along with enhanced non-photochemical quenching (NPQ) capacity, and metabolic reprogramming characterized by the accumulation of lipids, sugars, and starch alongside decreased protein yield. Metabolomics indicated reduced carbon supply for SCW polysaccharide biosynthesis, coupled with decreased protein yield and altered amino acid profiles characteristic of nitrogen-limited metabolism, which collectively favored the reallocation of carbon resources toward nitrogen-free high-value products. Transcriptomics confirmed the downregulation of SCW component biosynthetic genes and the upregulation of the methylerythritol phosphate (MEP) pathway and astaxanthin biosynthetic pathway. Scale-up experiments validated this strategy for producing astaxanthin-rich motile cells, offering a promising approach for microalgal biorefinery.

## 1. Introduction

Astaxanthin (3,3′-dihydroxy-β,β-carotene-4,4′-dione) is a natural ketocarotenoid renowned for its superior antioxidant capacity, attributed to its unique conjugated double-bond system and terminal hydroxyl and keto functional groups [1]. It has been shown that this pigment effectively scavenges free radicals and quenches singlet oxygen, ranking it among the most potent natural antioxidants [2]. In respiratory diseases, astaxanthin alleviates hyperoxia and lipopolysaccharide-induced lung injury associated with bronchopulmonary dysplasia in neonatal rats by enhancing antioxidant capacity, suppressing inflammatory factors, and reducing cell apoptosis [3]. In the field of metabolic diseases, astaxanthin improves insulin sensitivity and glucose tolerance in mice with gestational diabetes mellitus, with mechanisms involving the inhibition of reactive oxygen species production, regulation of IRS-1 phosphorylation, and promotion of GLUT4 membrane translocation [4]. In non-alcoholic fatty liver disease, astaxanthin ameliorates mitochondrial dysfunction and reduces hepatic lipid accumulation by activating the Nrf2/Keap1 pathway and upregulating FGF21/PGC-1α signaling [5,6]. Furthermore, astaxanthin restores mitophagy through activation of the PINK1-Parkin-p62-LC3 signaling pathway, reverses epithelial–mesenchymal transition, and thereby alleviates renal fibrosis [7]. In the field of oncology, astaxanthin induces G0/G1 phase cell cycle arrest in gastric cancer cells by inhibiting p-ERK and upregulating p27^kip-1^ expression [8]. In summary, astaxanthin exhibits promising pharmacological activities and drug development potential across various disease models through multi-target regulation of oxidative stress, inflammatory responses, and mitochondrial function.

Commercially, astaxanthin is produced through two routes: extraction from natural sources and chemical synthesis. However, synthetic astaxanthin, typically a racemic mixture of stereoisomers, is synthesized from petrochemical precursors and may contain residual chemical reagents, and is therefore not approved for direct human consumption [9,10]. Instead, its approved application is confined primarily to aquaculture, where it serves as a feed additive for pigmentation [9]. In contrast, natural astaxanthin from microalgae, predominantly in the (3S, 3′S) form, poses no such safety risks and exhibits superior bioactivity, making it the preferred focus of research and development [10]. In the marine environment, astaxanthin is a key carotenoid responsible for the characteristic pigmentation of various seafood, including salmon, shrimp, and krill, and it plays essential roles in their growth, reproduction, and stress resistance [11,12]. As such, natural astaxanthin is increasingly recognized as a high-value marine-derived nutraceutical with substantial applications in the pharmaceutical, food, and cosmetic industries [13,14].

Among natural sources, the green microalga *Haematococcus lacustris* (synonym: *H. pluvialis*) is recognized as a premier bio-factory for natural astaxanthin, capable of accumulating astaxanthin to over 3% of its dry cell weight under stress conditions, making it the most potent astaxanthin accumulator among all known organisms [15]. Although *H. lacustris* is typically found in freshwater and brackish habitats, it is also known to occur in coastal rock pools and other environments influenced by seawater intrusion, where fluctuating salinity and high light intensity represent natural selective pressures [12,16]. Thus, studying the adaptive mechanisms of *H. lacustris* under marine-relevant stressors (e.g., high salinity and high light) is of great significance for marine biotechnology and the sustainable production of marine bioactives. The life cycle of *H. lacustris* is complex, yet it can be broadly categorized into two main stages: a green motile stage and a red non-motile cyst stage. Industrial cultivation typically employs a two-stage strategy: first, promoting biomass accumulation of green motile cells under optimal conditions, followed by exposure to stress to induce cyst formation and astaxanthin biosynthesis [17]. High light intensity is the primary stressor, often applied synergistically with nitrogen deprivation or high salinity [18,19,20]. In addition to physical stressors, studies have demonstrated that the exogenous application of various chemical inducers can further enhance astaxanthin accumulation. These compounds, which include phytohormones (e.g., methyl jasmonate, gibberellin, and salicylic acid) [21,22], organic carbon sources (e.g., sucrose, and sodium citrate) [23,24], and amino acids (e.g., arginine, proline, and taurine) [25,26,27], act synergistically with stress conditions.

The accumulation of astaxanthin in *H. lacustris* under stress conditions is always accompanied by a morphological transition from motile cells to non-motile cysts, along with the formation of a thick and robust secondary cell wall (SCW) [28]. This physical barrier greatly complicates subsequent cell disruption for extraction and elevates processing costs, constituting a major bottleneck for the industrial-scale production of astaxanthin [29]. To address this limitation, research has been directed along two principal avenues: downstream cell disruption and upstream metabolic regulation. Downstream techniques, such as ionic liquid pretreatment and microbial fermentation-assisted disruption, are effective in breaking the cell wall [30,31,32]. However, these methods are frequently associated with high energy consumption, the need for specialized equipment, and potential introduction of impurities. In contrast, upstream strategies are focused on metabolic intervention. These approaches aim to cultivate improved algal strains that exhibit high astaxanthin yield and reduced cell wall thickness, which is regarded as a more promising and fundamental solution [33,34]. The exogenous application of specific chemicals offers a more convenient and efficient route for upstream regulation. Supplementation with taurine under high-light stress, or uridine under combined high-light and nitrogen-deprivation stress, promotes astaxanthin accumulation while simultaneously inhibiting SCW formation, yielding astaxanthin-rich motile cells that permit efficient extraction without mechanical disruption [27,35]. Although astaxanthin accumulation and SCW thickening are concomitant under stress, their biosynthetic pathways are not inherently coupled, thereby providing a theoretical basis for a “source reduction of cell wall thickness” breeding strategy.

Building on the evidence that nucleotide metabolism is linked to SCW formation, and that specific pyrimidines and purines can inhibit SCW formation [27,33,35,36,37], we further screened a series of nucleotides for their regulatory effects on both SCW formation and astaxanthin accumulation in *H. lacustris*. This screening identified cytidine monophosphate (CMP) as another nucleotide capable of effectively suppressing SCW formation, consistent with the emerging role of nucleotide derivatives in cell wall regulation [35]. However, CMP concurrently inhibited astaxanthin accumulation, thereby presenting a challenge for its standalone application. Based on this background, it was hypothesized that combining CMP with the astaxanthin-inducing stressor sodium chloride (NaCl) might concurrently suppress SCW formation and promote astaxanthin accumulation. This strategy might enable the efficient astaxanthin production in motile *H. lacustris* cells with reduced extraction barriers. This study evaluated the effects of combined CMP and NaCl application on astaxanthin yield and SCW formation. By identifying optimal concentrations, the impacts on cell growth, oxidative stress, and other physiological parameters were evaluated. Transcriptomic and metabolomic analyses were employed to elucidate the underlying mechanisms. Consequently, by demonstrating the efficacy of NaCl and CMP in generating astaxanthin-rich motile cells, this work lays a strategic and experimental groundwork for a more efficient astaxanthin production process, which is anticipated to facilitate downstream extraction and contribute to the development of marine microalgal biorefineries.

## 2. Results and Discussion

### 2.1. Effects of Combined NaCl and CMP Concentrations on Astaxanthin Yield and Motile Cell Proportion

To develop a strategy for enhancing astaxanthin accumulation while suppressing SCW formation in *H. lacustris*, cells were cultivated under high-light stress for 7 days with varying concentrations of NaCl and CMP. A two-way factorial analysis of variance (ANOVA) indicated that both factors, as well as their interaction, had highly significant effects on astaxanthin yield and the motile cell (without SCW) proportion (*p* < 0.0001), indicating that their influence is interdependent (Table 1). Compared to the unsupplemented control (CK), three combination treatments using 0.5 g/L NaCl with different CMP concentrations (0.25, 0.5, and 1.0 mM) significantly increased both astaxanthin yield and the motile cell proportion (Table 1). Among these, the combination of 0.5 g/L NaCl and 0.5 mM CMP (hereinafter, the NC group) yielded the highest motile cell proportion (96.8%) and the second-highest astaxanthin yield, resulting in the peak overall desirability score of 0.94 (see Section 3.4 for calculation). The astaxanthin yield of NC group was 35.6% higher than that of CK group. Therefore, the NC treatment is identified as the optimal condition for simultaneously promoting astaxanthin production and inhibiting SCW formation in *H. lacustris*.

The optimal NaCl concentration for astaxanthin accumulation in *H. lacustris* reported in the literature varies considerably among strains. Harker et al. found that 40 mM NaCl (~2.34 g/L) maximized volumetric astaxanthin yield in strain CCAP 34/7 [38], while Jin et al. reported that 5 g/L NaCl in a two-stage culture produced the highest astaxanthin yield in strain FACHB-712 [39]. Li et al. similarly demonstrated that 2 g/L NaCl applied during the induction stage markedly increased astaxanthin content in strain LUGU, while a low dose of 12.5 mg/L NaCl during the growth stage enhanced biomass [40]. Notably, Chekanov et al. reported that a newly isolated Arctic strain BM1 tolerated 25‰ salinity without adverse effects on growth, highlighting substantial strain-specific variability in salt responses [41]. In the present study, the 0.5 g/L NaCl treatment, despite being a much lower concentration than those typically reported, yielded the highest astaxanthin among all tested conditions. This low concentration aligns with the low-dose strategies reported in the literature. The synergistic combination of NaCl with CMP further amplifies this effect by simultaneously suppressing SCW formation, an outcome not achieved by NaCl alone in previous studies.

### 2.2. Effect of the Optimal Combination of NaCl and CMP on Cell Morphology and Size

**Figure 1:**
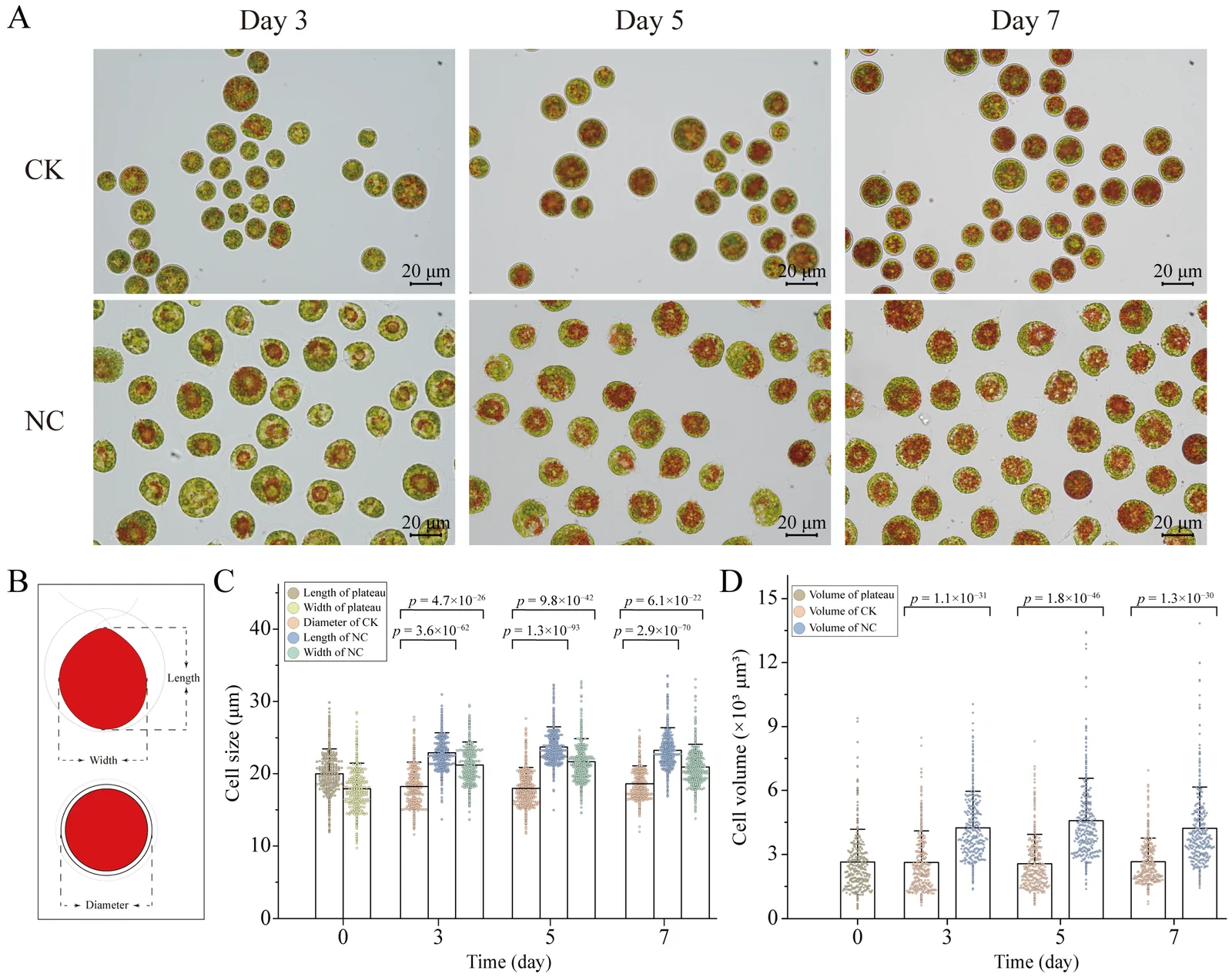
Effects of NaCl and CMP combination on cell morphology and cell size. (**A**) The phenotype of *H. lacustris* cells observed under optical microscope. (**B**) Schematic diagram of algal cell size measurement. (**C**) Cell diameter, length, and width; *n* = 300 biological replicates per group. (**D**) Cell volume, *n* = 300 biological replicates per group. NC: group treated with the combination of 0.5 g/L NaCl and 0.5 mM CMP; CK: untreated control group.

**Figure 2:**
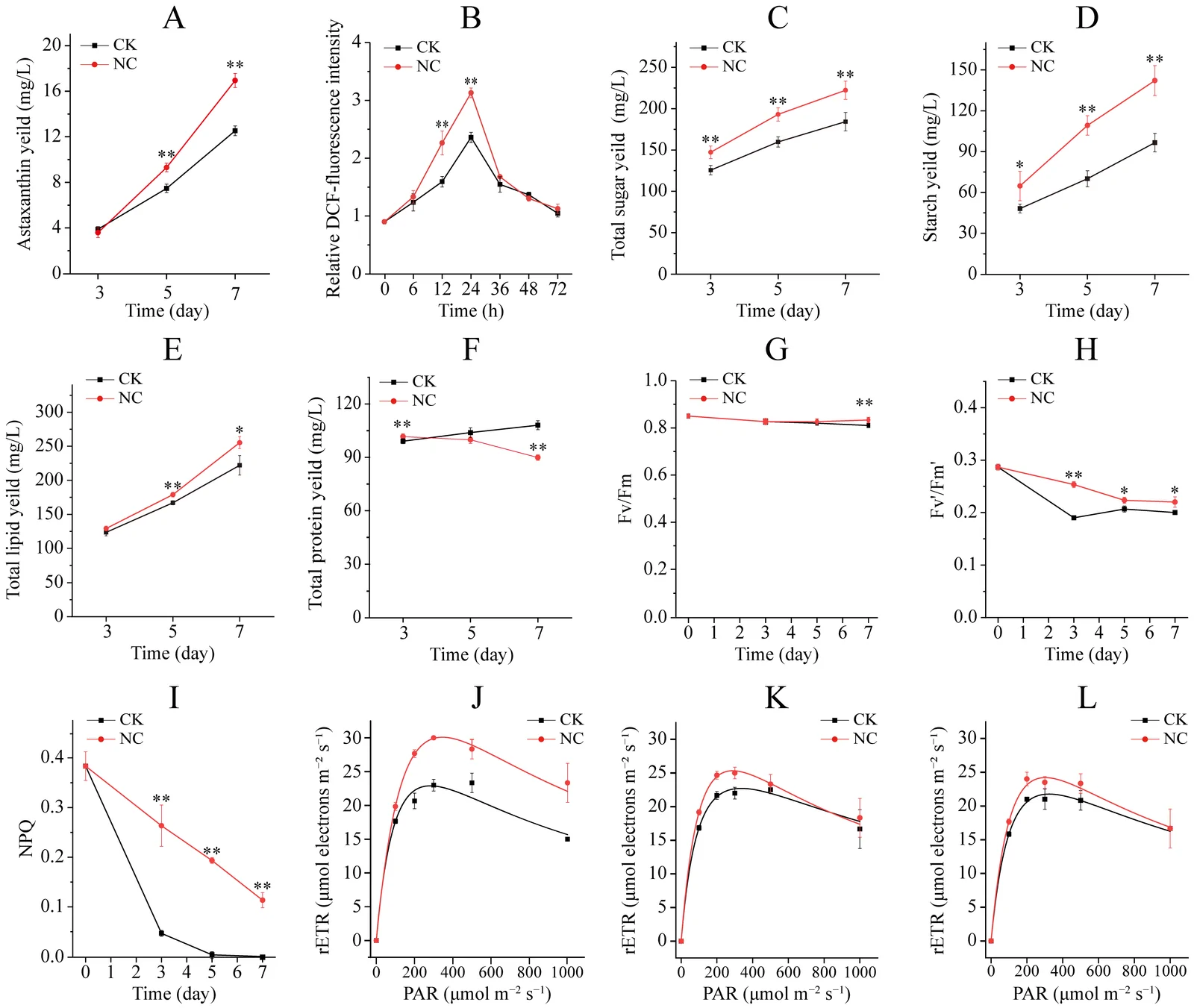
Effects of NaCl and CMP combination on physiological and biochemical indices of *H. lacustris* under high-light stress. (**A**) Astaxanthin yield in 3-L photobioreactor culture. (**B**) The relative abundance of reactive oxygen species (ROS). (**C**) Total sugar yields. (**D**) Starch yields. (**E**) Lipid yields. (**F**) Protein yields. (**G**) Maximum photochemical efficiency (Fv/Fm). (**H**) Effective photochemical efficiency (Fv′/Fm′). (**I**) Non-photochemical quenching (NPQ). (**J**–**L**) Relative electron transport rate II (rETR(II)) on days 3, 5, and 7, respectively. NC: group treated with the combination of 0.5 g/L NaCl and 0.5 mM CMP; CK: untreated control group. * and ** indicate significant (*p* < 0.05) and highly significant (*p* < 0.01) differences between NC and CK groups at the same time points. *n* = 3 biological replicates per group.

Under light microscopy, cells in both the NC and CK groups exhibited progressively deeper red coloration with prolonged high-light exposure (Figure 1A), with the NC group appearing markedly more intensely pigmented. These microscopic observations are consistent with the quantitative data reported in Table 1 and Figure 2A, where the NC treatment yielded significantly higher astaxanthin yield than the CK group. Prior to high-light stress (day 0), cells of both CK and NC groups were green and motile, with two flagella and no visible SCW (Figure S1). At each sampling time point, microscopic observation of independent culture aliquots revealed that cells in the CK group had progressively developed thick, transparent cell walls characteristic of SCW formation as defined by previously established light-microscopic criteria [27,35] and shed their flagella. On day 3, the majority of cells had become non-motile; by day 5, virtually all cells in the CK group had developed SCW. In contrast, even on day 7, the majority of cells in the NC group remained motile without apparent SCW development.

Furthermore, cells in the NC group appeared larger than those in the CK group (Figure 1A). Therefore, cell sizes were measured and compared. Motile cells without SCW typically exhibit an ovoid to pear-shaped morphology under light microscopy; thus, their length and width were measured (Figure 1B). Non-motile cells with SCW were spherical, so their diameters were measured. From day 3 to day 7, cell sizes within either the CK or NC group did not change significantly. However, both the length and width of NC group cells were significantly greater than the diameter of CK group cells (Figure 1C). The average cell length in the NC group was 23.26 μm, and the average width was 20.92 μm, whereas the average diameter of CK group cells was 18.59 μm. Given the distinct morphologies of the two cell types, linear dimensions alone provide an incomplete basis for size comparison. Cell volumes were therefore estimated using established geometric models (Section 3.2). On day 0, the mean cell volume was 2641 μm^3^. Following high-light exposure, the mean cell volume of the CK group remained relatively stable across days 3, 5, and 7, ranging from 2565 to 2660 μm^3^, with no significant difference compared to day 0 values (*p* > 0.05). In contrast, the NC group exhibited substantially larger volumes, with mean volume ranging from 4224 to 4579 μm^3^ across days 3, 5, and 7, all of which were significantly greater than the day 0 values (*p* < 0.001) and also significantly greater than those of the CK group at the corresponding time points (Figure 1D; *p* < 10^−30^). These findings confirmed that NC group cells were substantially larger by volume.

The linear dimension data are retained to enable comparison with published literature values, which in this field are predominantly reported as length, width, or diameter. The cell volumes observed in the present study are comparable to those reported for other *H. lacustris* strains. Chelebieva et al. reported mean zoospore volumes ranging from 1180 to 4159 μm^3^ across four strains, with the Black Sea strain IMBR-3 showing a mean volume of 2816 μm^3^ [42], close to the day 0 value (2641 μm^3^) in the present study. Notably, the volume response to stress also varied among strains in that study: IMBR-1 and CALU-79 showed little change in cell volume upon conversion to aplanospores, similar to our CK group, whereas IMBR-3 showed a 26% increase, and IMBR-2 showed a 268% increase. In the present study, the NC group cells exhibited an approximately 50% increase in volume compared to day 0 cells, which is within the range of strain-dependent variation documented by Chelebieva et al. [42]. These comparisons underscore that cell volume changes during stress are strongly influenced by strain-specific characteristics.

The cell sizes reported in this study are smaller than the dimensions typically documented for mature aplanospores in the literature. This is attributable to three factors. First, the extracellular matrix (ECM) was not included in our measurements. The ECM of *H. lacustris* is a transparent, gel-like layer that is optically invisible under bright-field microscopy and readily lost during sample preparation; its exclusion means our values reflect protoplast dimensions only [43]. Second, full maturation of aplanospores, including the development of a thickened secondary wall and the subsequent deposition of a tertiary wall, requires several weeks of sustained stress [44], whereas the present study applied only 7 days of high-light stress, yielding immature aplanospores. Third, substantial strain-dependent variation in cell size exists across *H. lacustris* isolates, and the values obtained here are consistent with those reported for comparable strains and conditions: Previous studies reported a control cell diameter of 22.3 ± 3.5 μm [43] and motile-derived cysts of 19.8–21.4 μm [45], which are close to our NC group and CK group, respectively. These morphological observations provide direct microscopic evidence corroborating the quantitative motile cell proportion data presented in Section 2.1.

### 2.3. Preliminary Scale-Up Validation of the Optimal Combination of NaCl and CMP in Promoting the Formation of Astaxanthin-Rich Motile Cells

To evaluate whether the combined NaCl and CMP treatment remains effective under increased culture volumes, the cultivation systems were scaled up 40-fold (from 60 mL in flasks to 2400 mL in a 3-L photobioreactor). The scale-up experiments (Figure 2A, Figures S2 and S3) were performed in a 3-L photobioreactor, while all other results presented in this study were obtained from 100 mL flask-scale cultures. The algal culture in the NC group appeared more intensely red compared to the CK group on day 7 (Figure S2). While no significant difference in astaxanthin yield was observed between the NC and CK groups on day 3 of high-light stress, the yield in the NC group was 30.4% and 37.0% higher than that of the CK group on days 5 and 7, respectively (Figure 2A). By day 7, nearly all the cells in the CK group had transformed into thick-walled non-motile cells, whereas almost all the cells in the NC group remained motile without developing SCW (Figure S3). These results indicate that the synergistic application of NaCl and CMP represents an effective upstream metabolic strategy for enhancing the co-production of astaxanthin and fatty acids while suppressing SCW formation in *H. lacustris*. This proof-of-concept at the 3-L scale provides a biological foundation for future process development, though further techno-economic evaluation and regulatory assessment would be required for industrial implementation.

Our laboratory has previously demonstrated that several nucleotides can inhibit SCW formation in *H. lacustris*, including uridine [35], and xanthosine [36]. The uridine–nitrogen-deprivation strategy requires centrifugation and resuspension of algal cells in nitrogen-free medium prior to stress induction, increasing operational complexity [35]. While xanthosine effectively suppresses SCW formation, it concomitantly inhibits astaxanthin accumulation, thereby compromising the primary objective of high-value product production [36]. By contrast, the NaCl and CMP combination developed here achieves robust SCW suppression and significant enhancement of astaxanthin and total fatty acid yields simultaneously, without requiring medium replacement. These advantages render the combination of NaCl and CMP strategy more operationally feasible for scaled-up production. Furthermore, NaCl is an inexpensive and readily available industrial chemical, and CMP is a commercially accessible nucleotide, making this combination economically viable. Our ongoing comparative studies also indicate that xanthosine combined with NaCl does not achieve comparable results to the NaCl and CMP combination in terms of either astaxanthin yield or motile cell proportion, suggesting that different nucleotides exert distinct effects on astaxanthin accumulation and SCW synthesis in *H. lacustris*.

### 2.4. Effect of the Optimal Combination of NaCl and CMP on Reactive Oxygen Species

Unfavorable conditions, such as high light, high temperature, nutrient deficiency, and salt stress, induce increased production of reactive oxygen species (ROS) in microalgae [46]. Excessive ROS can cause DNA damage and even lead to cell death [47]. Conversely, ROS also act as signaling molecules that upregulate key biosynthetic pathways for lipids and carotenoids in microalgae [48,49]. In *H. lacustris* specifically, chemical inducers have been shown to regulate ROS signaling to stimulate astaxanthin production under environmental stresses [50], and the MAPK signaling pathway has been proposed as a potential bridge connecting ROS signals to astaxanthin biosynthesis [51]. In the present study, the ROS levels, as indicated by H_2_DCFDA fluorescence intensity, in both the NC and CK groups increased rapidly under high-light stress, peaking at 24 h, and then gradually declined, returning to near-baseline levels by 72 h. Notably, at 12 h and 24 h, the ROS level in the NC group was 42% and 33% higher, respectively, than that in the CK group (Figure 2B). Despite this transient ROS elevation, the Fv/Fm remained stable throughout the 7-day stress period in both groups (Figure 2G), indicating that PSII reaction centers were not damaged by the oxidative burst. This pattern suggests that the elevated ROS in the NC group served primarily as a signaling cue rather than causing persistent photoinhibition, consistent with the role of ROS as signaling molecules in stress-induced carotenogenesis, and may have contributed to the higher astaxanthin accumulation observed in this treatment.

### 2.5. Effect of the Optimal Combination of NaCl and CMP on Accumulation of Biomass, Sugar, Starch, Lipid, and Protein

Under high-light stress, microalgae adjust their metabolic pathways to accumulate energy storage compounds, thereby enhancing their stress tolerance [52]. As shown in Figure S2 and Figure 2C–F, the dry weight, as well as the yields of total sugar, starch, and lipids in both the NC and CK groups increased with prolonged high-light exposure. However, while the dry weight and cell density showed no significant difference between the two groups at the same time points (Figure S4), the NC group exhibited significantly higher levels of total sugar, starch, and lipids compared to the CK group (Figure 2C–F). In the CK group, protein yield also rose over time, whereas in the NC group, it declined (Figure 2F). Although the protein level in the NC treatment was slightly higher than that in the CK group on day 3, it became significantly lower by day 7 (Figure 2F). In contrast, under high-light stress, the addition of NaCl alone initially promoted but later suppressed total sugar accumulation, increased lipid yield, and inhibited protein synthesis in *H. lacustris* [40]. These results suggest that CMP and NaCl act synergistically to alter the metabolic response pattern of *H. lacustris* under high-light conditions.

### 2.6. Effect of the Optimal Combination of NaCl and CMP on Chlorophyll Fluorescence Parameters

To investigate the combined effects of NaCl and CMP treatment on photosynthesis in *H. lacustris*, chlorophyll fluorescence parameters were measured at different time points. The variation trends of each indicator in the CK group were consistent with those reported by Liu et al. [36]. During 0–7 days of high-light stress, the maximum photochemical efficiency (Fv/Fm) of algal cells in both the NC and CK groups remained stable at approximately 0.85 (Figure 2G), which is close to the optimal Fv/Fm value reported for most plants [53,54]. This indicates that the structure of the PSII reaction center remained intact and undamaged in both groups despite the stress conditions. Notably, this value is higher than those reported for other *H. lacustris* strains under stress conditions [55,56]. However, consistent with this observation, relatively high Fv/Fm values were also observed in our recently published studies using the same strain (NBU489) under comparable high-light conditions [36,37]. These findings suggest that the high Fv/Fm values observed in the present study may reflect strain-specific photosynthetic characteristics and culture conditions.

High-light stress significantly reduced the actual photochemical efficiency (Fv′/Fm′) in both the NC and CK groups (Figure 2H). However, the Fv′/Fm′ values in the NC group were significantly higher than those in the CK group. This enhanced photochemical activity may provide more substrates and energy for material accumulation in the algal cells. The non-photochemical quenching (NPQ) values in both the NC and CK groups gradually decreased as high-light stress duration increased, with the NPQ in the NC group being significantly higher than that in the CK group (Figure 2I). This implies a stronger thermal dissipation capacity in the NC-treated algae. Furthermore, the relative electron transport rate II (rETR(II)) was higher in the NC group compared to the CK group, particularly on day 3 of high-light stress, indicating more efficient electron transport (Figure 2J–L). Jin et al. demonstrated that salinity stress in *H. lacustris* induces an excitation imbalance between PS II and PS I, with PS I-side electron transport being partially enhanced under certain saline conditions [39]. In the present study, the combination of NaCl and CMP may have amplified this PS I-side enhancement, contributing to the transient rETR increase. This interpretation is consistent with the higher ROS levels observed in the NC group (Figure 2B), as PSII/PSI imbalance is known to promote ROS generation, which in turn serves as a signaling cue for astaxanthin biosynthesis [39]. Nevertheless, the specific role of CMP in this process—whether through direct effects on photosynthetic phosphorylation, indirect modulation of nucleotide pools, or synergistic interaction with NaCl-induced stress signaling—remains unknown and merits further study.

The simultaneous elevation of Fv′/Fm′, rETR(II), and NPQ in the NC group may appear paradoxical, as enhanced NPQ typically reflects increased thermal dissipation of excitation energy. However, accumulating evidence indicates that photochemical efficiency and photoprotective capacity can increase concurrently in stress-acclimated photosynthetic organisms [57,58]. In this study, the higher ROS levels induced by the NC treatment (Figure 2B) likely triggered enhanced qE-type NPQ, which efficiently scavenged excess excitation energy. The sustained high Fv/Fm values and elevated Fv′/Fm′ in the NC group suggest that the increased NPQ was driven by the photoprotective qE component rather than the deleterious qI component [59]. Similar observations have been reported in high-light-acclimated plants displaying concomitantly higher NPQ and photosynthetic rates [60] and in microalgae *Fragilariopsis cylindrus* that upregulate NPQ while maintaining stable Fv/Fm under stress [61]. Collectively, these results demonstrate that the NaCl and CMP combination enabled effective photoacclimation in *H. lacustris* under high-light stress by maintaining robust photochemical activity alongside enhanced photoprotection.

### 2.7. Effect of the Optimal Combination of NaCl and CMP on Fatty Acid Yield

Under stress conditions, the accumulation of fatty acids in *H. lacustris* is markedly enhanced [62]. These fatty acids not only serve as energy reserves but also mitigate oxidative stress and support the coordinated synthesis of high-value metabolites such as astaxanthin, thereby improving the alga’s tolerance to adverse environments [63]. In humans, fatty acids, particularly polyunsaturated fatty acids (PUFAs), function as essential nutrients with demonstrated benefits including antioxidant and anti-inflammatory effects, as well as contributions to cardiovascular health, making them suitable for use in functional foods and health supplements [64].

To evaluate the impact of combined NaCl and CMP treatment on fatty acid production in *H. lacustris*, gas chromatography–mass spectrometry (GC-MS) analysis was conducted. A total of 21 fatty acids were identified, comprising six saturated fatty acids (SFAs), four monounsaturated fatty acids (MUFAs), and 11 PUFAs (Table 2). The most abundant fatty acids were palmitic acid (C16:0), linoleic acid (C18:2), linolenic acid (C18:3), hexadecatetraenoic acid (C16:4), and stearic acid (C18:0). In comparison with the CK group, the NC treatment significantly increased the yield of 15 individual fatty acids, whereas only two fatty acids (C16:2 and C20:3) showed significant decreases. Overall, the NC group exhibited higher yields of total fatty acids (TFAs, +28%), SFAs (+31%), MUFAs (+73%), PUFAs (+20%), and total UFAs (+25%) relative to the CK group. The fatty acid content per unit of dry weight (mg/g DW) was also compared (Table S1). Most of fatty acids in the NC group displayed higher contents than those in the CK group, consistent with the yield trends. In summary, the combined application of NaCl and CMP significantly promoted fatty acids accumulation in *H. lacustris* and enhanced its nutritional value.

### 2.8. Metabolic Profiling Reveals Reprogramming of Central Metabolism by the Optimal Combination of NaCl and CMP

**Figure 3:**
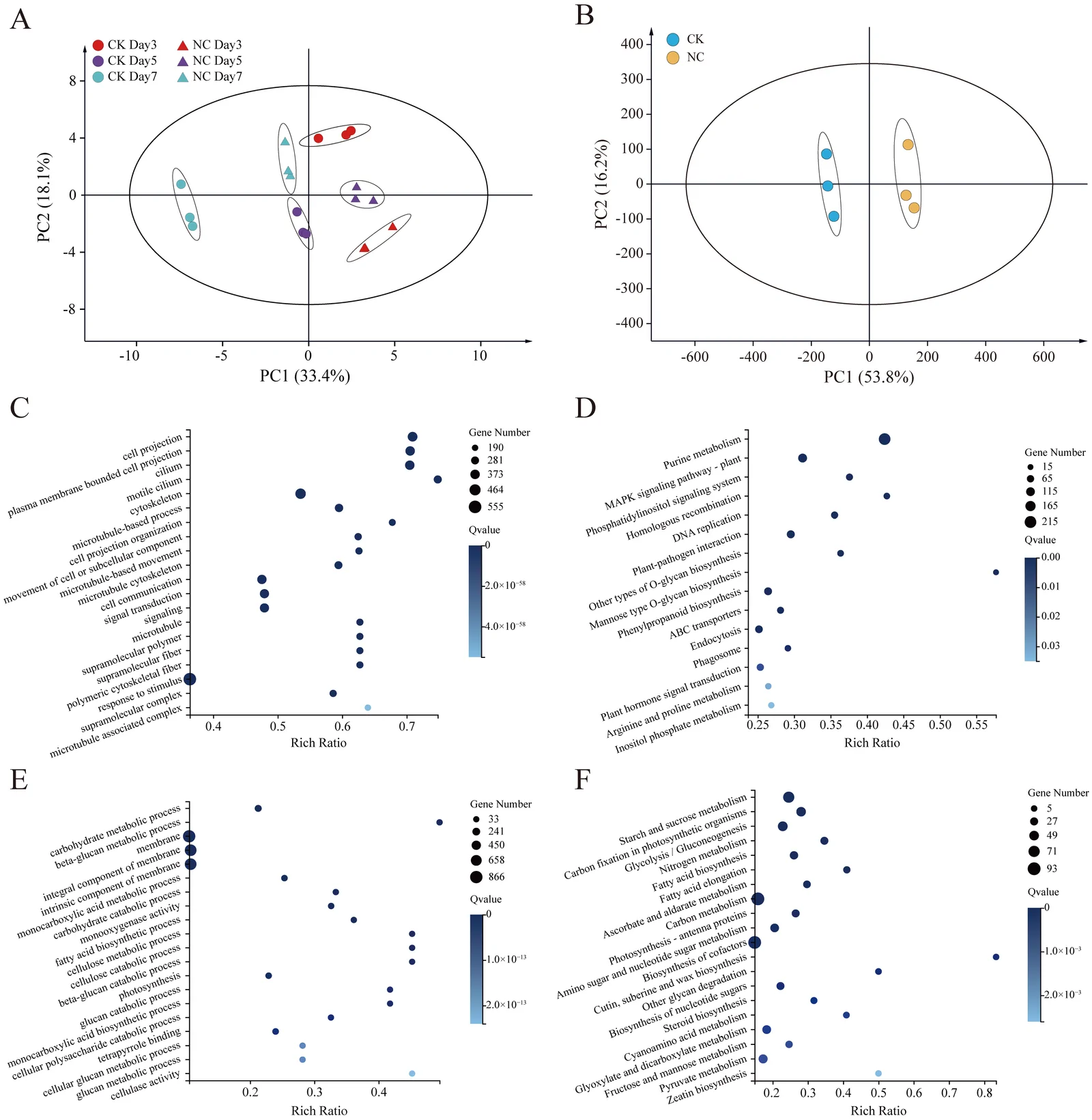
Overview of the effects of NaCl and CMP treatment on the metabolomic and transcriptomic changes in *H. lacustris*. (**A**) Principal component analysis (PCA) score plot of metabolomic data. (**B**) PCA score plot of transcriptomic data. (**C**) Enriched GO terms for up-regulated genes (URGs). (**D**) Enriched KEGG pathways for URGs. (**E**) Enriched GO terms for down-regulated genes (DRGs). (**F**) Enriched KEGG pathways for DRGs. NC: group treated with the combination of 0.5 g/L NaCl and 0.5 mM CMP; CK: untreated control group.

To elucidate the metabolic regulatory mechanisms underlying the effects of the combined NaCl and CMP treatment on *H. lacustris*, a comprehensive metabolomic analysis was performed using GC-MS on algal cells harvested on days 3, 5, and 7 of high-light stress. A total of 41 metabolites were identified, comprising 18 amino acids and their derivatives, 12 TCA-cycle and related organic acids, 4 sugars, and 7 other compounds (Table S2). Principal component analysis (PCA) was performed to get an overview of the metabolomic changes between NC and CK groups at different sampling time points. The first principal component (PC1), explaining 33.4% of total variance, showed a tendency to separate samples by cultivation time, and the second principal component (PC2), accounting for 18.1% of total variance, tended to separate NC from CK groups (Figure 3A), indicating that both cultivation time, and NC treatment have significant effect on metabolome of *H. lacustris* under high-light conditions. The three biological replicates for each group clustered relatively tightly in the PCA score plot (Figure 3A), suggesting high reproducibility and indicating that the observed differences between groups are more likely attributable to the treatment than to random biological or technical variation; this interpretation is further supported by the two-way ANOVA (Table 3). Compared to the CK group, 19, 23, and 16 differentially abundant metabolites were identified in the NC group on days 3, 5, and 7, respectively (Table 3). The two-way ANOVA revealed that the abundance of most differential metabolites was significantly influenced by the treatment, cultivation time, and their interaction (Table 3), which was consistent with the result of PCA.

A key finding was the consistent and significant reduction in the levels of specific sugars in the NC group. Notably, the content of mannose was significantly lower in the NC group than in the CK group across all three time points. Mannose is the precursor for mannan, which is the predominant polysaccharide in the SCW of *H. lacustris* and constitutes 89.4% of the total carbohydrate dry weight [44]. Furthermore, the levels of glucose and fructose were persistently lower in the NC group, and sucrose was significantly reduced on days 3 and 5. These results suggest a reduced carbon supply for cell wall polysaccharide biosynthesis, which is consistent with the observed inhibition of SCW formation.

The amino acid profile exhibited pronounced temporal dynamics. On day 3, the content of six amino acids, including tryptophan, serine, and cysteine, was significantly lower in the NC group. By day 5, the levels of several amino acids, such as serine, cysteine, and alanine, rebounded and even exceeded those in the CK group. However, by day 7, the content of seven amino acids was again significantly lower in the NC group. This pattern aligns with the observed decrease in total protein yield in the NC group (Figure 2F) and implies that the combined treatment of NaCl and CMP may induce a nitrogen-deficient state. This nitrogen-limited metabolic signature, characterized by reduced protein and amino acid levels alongside altered carbohydrate and fatty acid profiles, is supported by metabolomic studies of nitrogen-starved *H. lacustris*: Recht et al. reported that nitrogen deprivation triggers sequential accumulation and subsequent degradation of carbohydrates concomitant with fatty acid synthesis [65], while Recht et al. also demonstrated a sharp decline in protein (from ~28% to ~7% dry weight) and amino acid levels as carbon is redirected toward fatty acid and astaxanthin accumulation [66]. Salinity stress is known to inhibit nitrate reductase activity, a key enzyme in nitrogen assimilation [67]. This metabolic pressure is consistent with a potential shift in nitrogen and carbon utilization from protein synthesis toward high-value product accumulation like astaxanthin and fatty acids, thereby optimizing resource allocation under stress [37,68,69].

The levels of most TCA-cycle and related organic acids, including citric acid and malic acid, were lower in the NC group. This suggests an accelerated utilization of tricarboxylic acid (TCA) cycle intermediates, potentially to supply energy and carbon precursors for the enhanced biosynthesis of astaxanthin and fatty acids observed in the NC treatment.

### 2.9. Impact of Optimal Combination of NaCl and CMP on the Transcriptome of H. lacustris

#### 2.9.1. Overview of Transcriptomic Differences

To study the mechanism by which the optimal combination of NaCl and CMP affects the growth and physiological changes in *H. lacustris*, the transcript levels of genes were analyzed by RNA-seq analysis on algal cells from both the NC and CK groups after two days of high-light exposure. The analysis produced an average of 21.6 million clean reads per sample. In total, 43,752 genes were annotated by mapping the reads to the reference genome of *H. lacustris* [70]. PCA revealed clear transcriptional differences between the NC and CK groups, with PC1 accounting for 53.8% of the total variance, distinctly separating the two groups (Figure 3B). This result indicates that the combination of NaCl and CMP significantly altered the transcriptome of *H. lacustris* under high-light stress.

Compared to the CK group, the NC group exhibited 13,364 differentially expressed genes (DEGs), with 8591 upregulated genes (URGs) and 4773 downregulated genes (DRGs). To gain a systemic insight into the biological processes and pathways involved, the DEGs were subjected to Gene Ontology (GO) and Kyoto Encyclopedia of Genes and Genomes (KEGG) pathway enrichment analyses. A total of 262 GO terms were significantly enriched for URGs, categorized into 125 “biological processes”, 47 “cellular components”, and 90 “molecular functions” (Table S3). The top of enriched GO terms was related to flagella, cytoskeleton, microtubule, and cell movement (Figure 3C), aligning with the observed presence of flagella and motility in NC group cells and their absence in CK group cells. Fifteen KEGG pathways were significantly enriched for URGs, with the top three being purine metabolism, MAPK signaling (plants), and phosphatidylinositol signaling system (Figure 3D).

In contrast, 307 GO terms were significantly enriched for DRGs, categorized into 172 “biological processes”, 34 “cellular components”, and 101 “molecular functions” (Table S4). The top of enriched GO terms was associated with carbohydrate metabolic process, glucan metabolic process, cellulose metabolic process, and component of membrane, etc. (Figure 3E). The downregulation of glucan and cellulose biosynthesis was consistent with the observed phenotype that SCW was formed in the CK group but was not formed in the NC group. A total of 27 KEGG pathways were significantly enriched, including starch and sucrose metabolism, nitrogen metabolism (Figure 3F and Table S5). The downregulation of nitrogen metabolism may contribute to the decrease in total protein production in the NC group.

#### 2.9.2. Biosynthetic Pathways of Secondary Cell Wall Components

**Figure 4:**
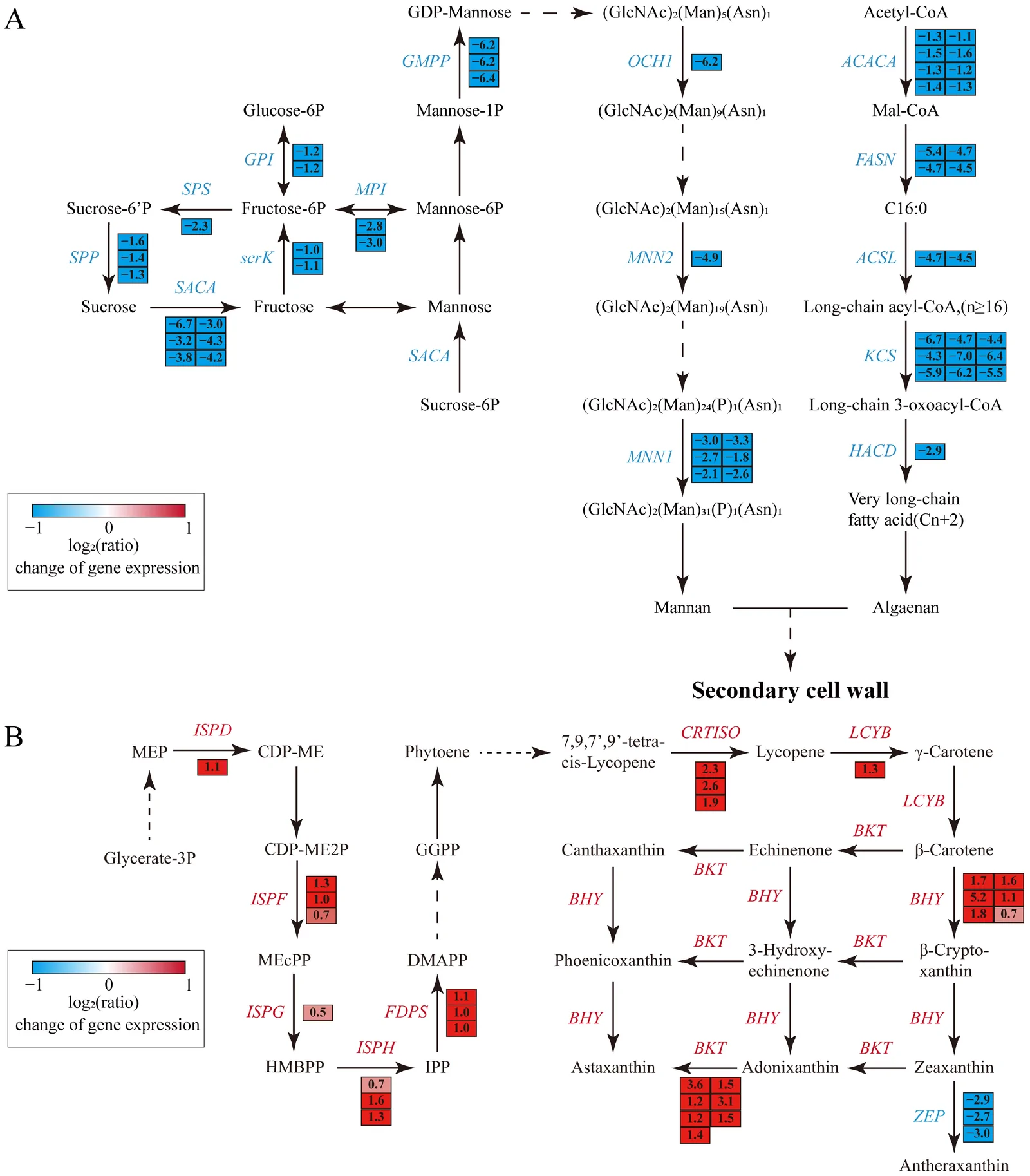
Effects of NaCl and CMP combination on the expression of genes involved in biosynthesis of SCW components (**A**), and the MEP and astaxanthin biosynthetic pathways (**B**). The values in each cell beside the gene indicate the log_2_-transformed gene expression ratio between the NC and CK groups. Red and blue of the cells indicate genes significantly up-regulated and down-regulated in the NC group compared with the CK group, respectively. Solid lines indicate continuous enzymatic steps, while dashed lines represent multi-step biochemical reactions. ACACA, acetyl-CoA carboxylase; ACSL, long-chain acyl-CoA synthetase; BHY, beta-carotene hydroxylase; BKT, beta-carotene ketolase; CDP-ME, 4-(cytidine 5′-diphospho)-2-C-methyl-D-erythritol; CDP-ME2P, 2-phospho-4-(cytidine 5′-diphospho)-2-C-methyl-D-erythritol; CRTISO, prolycopene isomerase; DMAPP, dimethylallyl diphosphate; FASN, fatty acid synthase; FDPS, farnesyl diphosphate synthase; GGPP, geranylgeranyl pyrophosphate; GMPP, mannose-1-phosphate guanylyltransferase; GPI, glucose-6-phosphate isomerase; HACD, 3-hydroxyacyl-CoA dehydrogenase; HMBPP, 1-hydroxy-2-methyl-2-butenyl 4-diphosphate; IPP, isopentenyl diphosphate; ISPD, 2-C-methyl-D-erythritol 4-phosphate cytidylyltransferase; ISPF, 2-C-methyl-D-erythritol 2,4-cyclodiphosphate synthase; ISPG, 4-hydroxy-3-methylbut-2-enyl-diphosphate synthase; ISPH, 4-hydroxy-3-methylbut-2-enyl diphosphate reductase; KCS, 3-ketoacyl-CoA synthase; LCYB, lycopene beta-cyclase; MEcPP, 2-C-methyl-D-erythritol 2,4-cyclodiphosphate; MEP, methylerythritol 4-phosphate; MNN1, alpha-1,3-mannosyltransferase; MNN2, alpha-1,2-mannosyltransferase; MPI, mannose-6-phosphate isomerase; OCH1, alpha-1,6-mannosyltransferase; SACA, sucrose-6-phosphate hydrolase; scrK, fructokinase; SPP, sucrose phosphatase; SPS, sucrose-phosphate synthase; ZEP, zeaxanthin epoxidase.

Given that the combined treatment of NaCl and CMP significantly inhibited SCW formation, the expression levels of genes involved in biosynthesis of SCW components were analyzed (Figure 4A). The biosynthesis of mannan is catalyzed by mannosyltransferases using GDP-mannose as a substrate [36,37,71]. In the NC treatment group, the expression of multiple genes encoding enzymes critical for GDP-mannose synthesis was significantly downregulated. These enzymes include sucrose-6-phosphate hydrolase (SACA), fructokinase (scrK), glucose-6-phosphate isomerase (GPI), sucrose-phosphate synthase (SPS), sucrose phosphatase (SPP), mannose-6-phosphate isomerase (MPI), and mannose-1-phosphate guanylyltransferase (GMPP). Furthermore, the expression of several genes within the mannosyltransferase family, essential for polymer elongation, was also markedly suppressed. These included genes encoding alpha-1,6-mannosyltransferase (OCH1), alpha-1,2-mannosyltransferase (MNN2), and alpha-1,3-mannosyltransferase (MNN1). Algaenan, a highly resistant and non-hydrolyzable aliphatic biopolymer, is a key component contributing to the robustness of the microalgal SCW. Concurrently, the algaenan biosynthetic pathway was also significantly affected. The expression of genes encoding key enzymes such as acetyl-CoA carboxylase (ACACA), fatty acid synthase (FASN), long-chain acyl-CoA synthetase (ACSL), 3-ketoacyl-CoA synthase (KCS), and 3-hydroxyacyl-CoA dehydrogenase (HACD) was significantly downregulated in the NC group. In conclusion, the combination of NaCl and CMP inhibits SCW formation in *H. lacustris* by downregulating the expression of critical genes involved in the biosynthesis of both the polysaccharide mannan and the aliphatic polymer algaenan, thereby leading to an increased proportion of motile cells.

#### 2.9.3. Astaxanthin Biosynthetic Pathway

The combined treatment of NaCl and CMP significantly promoted astaxanthin accumulation. Accordingly, the expression levels of genes involved in the methylerythritol 4-phosphate (MEP) pathway and the astaxanthin biosynthetic pathway were analyzed (Figure 4B). The MEP pathway supplies isopentenyl pyrophosphate (IPP) and dimethylallyl pyrophosphate (DMAPP), the precursors for geranylgeranyl pyrophosphate (GGPP), which is the substrate for astaxanthin synthesis. In this pathway, the expression of genes encoding 2-C-methyl-D-erythritol 4-phosphate cytidylyltransferase (ISPD), 2-C-methyl-D-erythritol 2,4-cyclodiphosphate synthase (ISPF), 4-hydroxy-3-methylbut-2-enyl diphosphate reductase (ISPH), and farnesyl diphosphate synthase (FDPS) was significantly up-regulated in the NC group. In the astaxanthin synthesis pathway, the expression of genes encoding prolycopene isomerase (CRTISO), lycopene beta-cyclase (LCYB), beta-carotene ketolase (BKT), and beta-carotene hydroxylase (BHY) was also significantly up-regulated in the NC group compared to the CK group. In contrast, the expression of the genes encoding zeaxanthin epoxidase (ZEP), which catalyzes the conversion of zeaxanthin into antheraxanthin and violaxanthin, was significantly down-regulated in the NC group. These results indicate that the combination of NaCl and CMP promotes astaxanthin accumulation in *H. lacustris* not only by up-regulating key enzyme genes in the MEP and astaxanthin biosynthetic pathways, but also by down-regulating *ZEP* expression, thereby limiting the diversion of zeaxanthin away from both photoprotective and astaxanthin precursor pools.

Because ZEP mediates the epoxidation of zeaxanthin back to violaxanthin [72], its transcriptional down-regulation is consistent with zeaxanthin retention. Accumulated zeaxanthin may contribute to photoprotection via two non-mutually exclusive routes. First, zeaxanthin is known to potentiate qE-type non-photochemical quenching (NPQ), acting either as a direct quencher of excited chlorophyll or as an allosteric modulator of antenna protein conformation [73,74]. Second, the retained zeaxanthin may also serve as a direct precursor for astaxanthin synthesis via ketolation reactions catalyzed by BKT (Figure 4B), thereby coupling photoprotection with secondary carotenoid accumulation.

In higher plants, qE is predominantly governed by the PsbS protein and the violaxanthin cycle [59,75]. In *H. lacustris*, transcriptomic analyses have revealed the expression of both PsbS and LhcsR (light-harvesting complex stress-related) proteins, with their transcripts transiently up-regulated during early stress exposure, while the expression of violaxanthin cycle enzyme genes remains relatively stable [76]. These observations suggest that, unlike in higher plants, the violaxanthin cycle may play a secondary role in modulating NPQ in *H. lacustris*, where PsbS and LhcsR appear to be more prominently involved [76]. Nevertheless, it should be noted that these conclusions are primarily derived from transcriptomic surveys, and direct functional validation of these proteins in NPQ regulation remains constrained due to the technical challenges of genetic manipulation in *H. lacustris*. Therefore, while we interpret our findings within the broader framework of qE photoprotection established in well-characterized systems [59,61], we acknowledge that the specific molecular players and their relative contributions may differ across species.

#### 2.9.4. Nucleotide Metabolism

Comprehensive pathway analysis revealed a pronounced transcriptional activation in the purine and pyrimidine metabolic pathways of *H. lacustris* under the combined NaCl and CMP treatment, evidenced by the substantial up-regulation of genes within these pathways (Figures S5 and S6). Specifically, in the purine metabolism pathway, 215 out of 240 (89.6%) DEGs were significantly up-regulated in the NC group, whereas only 25 genes were down-regulated (Table S6). This transcriptional enhancement may reflect the critical functions of purine nucleotides as energy carriers and signaling molecules in stress adaptation processes [77]. A similar trend was noted in the pyrimidine metabolism pathway, where 32 out of 34 (94.1%) DEGs were up-regulated, and only two genes were down-regulated (Table S7). In accordance with consistent observations from previous studies on *H. lacustris* under high-light stress, where inhibition of SCW formation is invariably associated with up-regulated expression of nucleotide metabolism pathway genes [27,33,35,36], the present study under NC treatment demonstrated a concurrent up-regulation of nucleotide metabolism pathway genes, suppression of SCW component biosynthetic genes, and inhibition of SCW formation. However, the direct regulatory effect of nucleotide metabolism on SCW formation requires further investigation, for instance, through approaches such as exogenous addition of nucleotide inhibitors or genetic manipulation to reduce nucleotide biosynthesis, in order to observe subsequent changes in SCW formation in *H. lacustris*.

It is worth noting that the transcriptomic data (Day 2) and metabolomic data (Days 3–7) were collected at different time points to capture early transcriptional responses and subsequent metabolite accumulation, respectively. While both datasets independently support the conclusion that the NC treatment may redirect carbon flux toward astaxanthin and fatty acid production while suppressing SCW formation, this temporal offset should be considered when interpreting the consistency between gene expression and metabolite levels.

## 3. Materials and Methods

### 3.1. Algal Culture and Treatment

*Haematococcus lacustris* (synonym: *H. pluvialis*) NBU489 was obtained from a laboratory culture collection. It was grown in a sterilized NBU3^#^ basal medium for a two-stage cultivation [27]. The NBU3^#^ basal medium contained the following components: NaNO_3_ (100 mg/L), Na_2_EDTA (20 mg/L), K_2_HPO_4_ (10 mg/L), FeSO_4_·7H_2_O (2.5 mg/L), MnSO_4_ (0.25 mg/L), vitamin B_12_ (5 × 10^−4^ mg/L), and vitamin B_1_ (1.2 × 10^−3^ mg/L). The pH of NBU3^#^ basal medium was 7.0–7.5 prior to autoclaving. Photon flux density (PFD) was measured using a wireless spectral illuminance meter (HPCS-330P, HOPOOCOLOR, Hangzhou, China). The light intensity at the culture surface level was adjusted and verified with this meter prior to and during the experiments. In the initial vegetative growth phase, cells were inoculated at an initial density of approximately 2.2 × 10^4^ cells mL^−1^ and cultured under low-light intensity (25–30 μmol m^−2^ s^−1^) provided by cool white LED lamps with a 12 h:12 h light-dark cycle at 24 °C for 14 days, until reaching the early stationary phase with a cell density of approximately 2.2 × 10^5^ cells mL^−1^. The culture was then thoroughly mixed to ensure homogeneity, and aliquots were dispensed into separate flasks for different treatment groups. Subsequently, during the stress induction phase, algal cultures were directly transferred, without replacing the medium, to continuous high-light intensity (~200 μmol m^−2^ s^−1^, provided by the same type of LED lamps) at 24 °C to stimulate astaxanthin accumulation. For flask-scale experiments, the cultures were manually shaken six times daily to facilitate gas exchange and prevent cell aggregation, without forced aeration.

Unless otherwise specified, early stationary-phase cells were inoculated into 100 mL conical flasks containing 60 mL of culture medium for astaxanthin induction. A two-factor orthogonal design was employed to investigate the effects of sodium chloride (NaCl; concentrations: 0, 0.25, 0.50, and 1.00 g/L) and cytidine monophosphate (CMP; concentrations: 0, 0.25, 0.50, and 1.00 mM). On day 7 post high-light exposure, samples were collected to determine the proportion of motile cells and astaxanthin yield. Based on these results, the optimal concentration combination was identified, and algal cells treated with this combination in flask cultures were further sampled on days 3, 5, and 7 for analysis of starch, total sugar, lipid, and protein yields, as well as for morphological observations.

For scale-up experiments, 2400 mL of stationary-phase algal culture was transferred to a 3 L photobioreactor. To compensate for the increased optical path length and light attenuation associated with the larger working volume, the light intensity was increased to 400 μmol m^−2^ s^−1^ to ensure sufficient light penetration for effective astaxanthin induction. The optimal combination of NaCl and CMP was added to the experimental group, while the control group remained untreated. The cultures were incubated at 24 °C under continuous high-light intensity (400 μmol m^−2^ s^−1^) with aeration of filtered air at a flow rate of 0.2 vvm (volume of air per volume of medium per minute).

### 3.2. Quantification of Astaxanthin Yield

Astaxanthin yield was measured spectrophotometrically as described by Zhang et al. [27] with minor modifications. A 3 mL algal culture was centrifuged, and the pellet was resuspended in 6 mL dimethyl sulfoxide (DMSO) for extraction at 65 °C for 20 min. After centrifugation at 3000× *g* for 5 min, absorbance of the supernatant was measured at 530 nm using a TU-1810Plus UV–Vis spectrophotometer (XIPU, Shanghai, China) with glass cuvettes (1.0 cm optical path length). The standard curve was constructed using commercial astaxanthin (≥98% purity, Solarbio, Beijing, China) dissolved in DMSO over the concentration range of 0–40.00 mg/L. The linear equation was C (mg/L) = 8.2184 × A_530_ (R^2^ = 0.9999).

### 3.3. Assessment of Motile Cell Proportion and Cell Size

For each sample, 1 mL of algal culture was centrifuged, and the supernatant was carefully removed, leaving approximately 30 μL of liquid to resuspend the cell pellet by pipetting. After thorough mixing, 10 μL of the concentrated cell suspension was transferred onto a glass slide and covered with a coverslip for microscopic observation. Morphological observation of algal cells was performed at 400× magnification using a light microscope equipped with a digital camera (ToupCamTM, Touptek, Hangzhou, China). The identification of SCW-forming cells (non-motile cysts) was based on previously validated light-microscopic criteria: SCW-forming cells are characterized by spherical morphology and the presence of a distinct, transparent cell wall, whereas motile cells lacking SCW appear oval-shaped without a visible SCW. These morphological criteria have been validated in our previous studies by RCA120 fluorescence staining, which labels the mannan components of the SCW [27,35], and by transmission electron microscopy (TEM) [35]. For assessment of motile cell proportion, at least five random fields were imaged at 200× magnification per replicate. The number of motile (N1) and non-motile (N2) cells were manually counted. Motile cells were identified as oval-shaped cells lacking a visible SCW, while non-motile cells were recognized by their spherical morphology and the presence of a distinct, transparent SCW. The proportion of motile cells was calculated as N1/(N1 + N2), where N1 + N2 represents the total number of cells counted per sample, maintained at approximately 200 cells to ensure statistical reliability.

Subsequently, the same set of images was used for cell size measurement. The images were imported into ImageJ software (version 1.53e, National Institutes of Health, Bethesda, MD, USA) and calibrated using the scale bar embedded during capture. The cell size was then measured for 100 cells per replicate (i.e., 300 cells per group across three biological replicates). The length and width were measured for motile cells, while the diameter was measured for non-motile cells. Cell volumes were approximated using geometric models according to Chelebieva et al. [42]. CK group cells (spherical) were modelled as spheres: V = πd^3^/6, where d was the cell diameter. NC group cells (ovoid to pear-shaped) were modelled as prolate spheroids: V = (4/3)πab^2^, where a = length/2 and b = width/2. Individual cell volumes were calculated from each measured length and width (or diameter) value, and the mean volume was then computed for each biological replicate. These geometric models approximate idealized cell shapes and may not fully capture the natural morphological variation of individual cells; therefore, the calculated volume values should be interpreted as estimates rather than absolute measurements.

### 3.4. Analysis of the Optimal Concentration Combination

To identify the optimal combination of NaCl and CMP concentrations, the data from the 16 treatment combinations shown in Table 1 were statistically analyzed using Design-Expert software (version 13, Stat-Ease, Minneapolis, MN, USA), with astaxanthin yield and motile cell proportion as the two response variables. The Factorial-Randomized-Multilevel Categoric design was selected, with the process order set to 2Fl (second-order factor effects). After performing analysis of variance (ANOVA) separately for each response variable, the desirability function was applied. Both response variables (astaxanthin yield and motile cell proportion) were assigned equal weight in the desirability calculation. Equal weights were assigned because no standardized weighting criteria have been established for these two responses in *H. lacustris* cultivation optimization, and both parameters are equally critical for achieving the dual objective of maximizing high-value product accumulation while minimizing the SCW barrier. The desirability (d) for each variable was calculated as: d = (A − B)/(C − B), where A is the measured value, B is the minimum response across all groups, and C is the maximum response value. Here, d_1_ represents the desirability for astaxanthin yield, and d_2_ for the proportion of motile cells. The overall desirability (D) was derived as the geometric mean of the individual desirabilities: D = (d_1_ × d_2_)^1/2^. Based on this method, the treatment combination of 0.5 g/L NaCl and 0.5 mM CMP was identified as the optimal condition, yielding the highest overall desirability score (D = 0.94). To verify the robustness of this weighting scheme, we performed additional calculations using alternative weight ratios (astaxanthin yield:motile cell proportion = 3:2 and 2:1). Under both scenarios, the optimal combination remained 0.5 g/L NaCl and 0.5 mM CMP with the highest desirability score, confirming that the identified optimal condition is not sensitive to the specific weight allocation. This method follows the standard desirability function approach for multi-response optimization described by Myers and Montgomery (Response Surface Methodology) and implemented in Design-Expert software. For details, see https://statease.com/docs/v23.1/contents/optimization/desirability-details/ (accessed on 10 January 2026).

### 3.5. Determination of Cell Density and Dry Weight

Cell density was monitored using a plankton counting chamber under an optical microscope. Briefly, 100 μL of algal culture was loaded onto the counting chamber, and cells were counted in five different visual fields. Each sample was measured three times with different visual windows, and the average was calculated. To determine the dry weight (DW) of algal biomass, 25 mL of algal culture was centrifuged to pellet the cells. The cell pellet was washed twice with distilled water to remove residual medium and salts, followed by freeze-drying for 48 h using a freeze dryer (SCIENTZ-10N/A, SCIENTZ, Ningbo, China) to remove moisture before being weighed on an analytical balance. Three biological replicates were prepared for each treatment.

### 3.6. Measurement of ROS Level

Reactive oxygen species (ROS) were quantified using the fluorescent probe 2′,7′-dichlorodihydrofluorescein diacetate (H_2_DCFDA, Biosharp, Beijing, China) according to the methods described previously [23,78]. Cells were sampled at 0–72 h under high-light, washed with phosphate-buffered saline (2.0 mM KH_2_PO_4_, 137 mM NaCl, 10.0 mM Na_2_HPO_4_, 2.7 mM KCl, pH 7.4), and incubated with 10 μM H_2_DCFDA at 37 °C for 30 min in the dark. Fluorescence intensity was measured with a multifunctional microplate reader (Molecular Devices, San Jose, CA, USA) at excitation and emission wavelengths of 488 nm and 525 nm, respectively.

### 3.7. Measurement of Starch Yield

Starch yield was analyzed using a Plant Starch Content Assay Kit (Keming, Suzhou, China) according to manufacturer’s instructions as outlined by Liu et al. [36].

### 3.8. Measurement of Total Sugar Yield

Total sugar yield was determined with a Total Sugar Content Assay Kit (Keming, Suzhou, China) in accordance with the manufacturer’s protocol, as previously described by Liu et al. [36].

### 3.9. Analysis of Lipid Yield

The lipid yield was determined according to the method described by Xing et al. [37] with modifications. Total lipids from 60 mL cultures were extracted with 1 mL of chloroform-methanol (2:1 *v*/*v*) under agitation at 25 °C for 10 min, repeated until the residue turned grayish-white. Extracts were evaporated to constant weight at 37 °C. Lipid yield (C_lipid_, mg/L) was calculated as: C_Lipid_ = [(M2 − M1)/60] × 1000, where M1 and M2 are the weights of the aluminum dish before and after extraction, respectively.

### 3.10. Determination of Protein Yield

Total protein yield was measured using the Bradford protein assay (Coomassie Brilliant Blue G-250) according to Li et al. [79] with minor modifications. Algal cells from 10 mL of culture were harvested by centrifugation, and the supernatant was discarded. The cell pellet was ground with a mortar and pestle, and then resuspended in 200 μL of 1 M NaOH solution. The suspension was incubated in an 80 °C water bath for 10 min, followed by the addition of 800 μL of distilled water and thorough mixing. After centrifugation at 12,000× *g* for 10 min, the supernatant was transferred to a new tube. This extraction procedure was repeated once, and the two supernatants were pooled to obtain a final volume of approximately 2 mL of protein extract. An aliquot of 0.4 mL of the extract was taken and diluted five-fold with distilled water. Then, 10 mL of Coomassie Brilliant Blue G-250 solution was added to the diluted sample, and the mixture was allowed to react for 10 min at room temperature. The absorbance was measured at 595 nm (A_595_) using a TU-1810Plus UV–Vis spectrophotometer (XIPU, Shanghai, China). The standard curve was prepared using bovine serum albumin (Macklin, Shanghai, China) dissolved in 12.5 mM NaOH aqueous solution over the concentration range of 0–75 mg/L. The linear equation was C(mg/L) = 99.69 × A_595_ (R^2^ = 0.9795).

### 3.11. Measurement of Chlorophyll Fluorescence Parameters

Chlorophyll fluorescence parameters were evaluated using a PSI AquaPen AP 110-C fluorometer (Photon System Instruments, Drásov, Czech Republic) on days 0, 3, 5, and 7 after high-light exposure. The instrument was equipped with blue LEDs (peak wavelength 455 nm). For each measurement, 2 mL of fresh algal culture was transferred into the manufacturer-supplied cuvette. The measuring light (Flash pulse = 10%; ~0.009 μmol photons m^−2^ per pulse), saturating pulse (Super pulse = 100%; ~3000 μmol photons m^−2^ s^−1^), and actinic light (Actinic pulse = 200 μmol photons m^−2^ s^−1^) settings were used, with all other parameters following the manufacturer’s default protocols.

Maximum PSII quantum yield in the dark-acclimated state (Fv/Fm) and light-acclimated state (Fv′/Fm′), as well as non-photochemical quenching (NPQ), were measured using the NPQ1 protocol. After 15 min of dark adaptation, the NPQ1 measurement mode was initiated. The NPQ1 protocol applies a sequence of five saturating pulses during actinic light exposure (60 s per phase), followed by three dark recovery phases. Fv/Fm corresponds to QY_max (dark-adapted), Fv′/Fm′ corresponds to QY_Lss, and NPQ corresponds to NPQ_Lss, all of which were calculated based on light-adapted steady-state data (Fm_Lss and Ft_Lss). The instrument software automatically marked the data from the last (fifth) saturating pulse of the light phase as the steady-state parameters. The Fv/Fm, Fv′/Fm′, and NPQ values were automatically calculated and directly exported by the instrument software, with no manual calculation required.

For rETR(II), the LC2 protocol was used with the instrument’s default settings. Actinic light intensities of 100, 200, 300, 500, and 1000 μmol photons m^−2^ s^−1^ were applied sequentially. For each light intensity (PAR), the corresponding QY_par_ value was automatically recorded. The rETR_par_ value for each light intensity was then calculated as rETR_par_ = QY_par_ × PAR × 0.5, where PAR denotes the actinic light intensity. The final rETR(II) curves were generated by fitting the calculated rETR_par_ values against the corresponding PAR values using the equation rETR(II) = PAR/(a·PAR^2^ + b·PAR + c) [80] via nonlinear regression using OriginPro 2026 software. All measurements were performed with three biological replicates.

### 3.12. Analysis of Fatty Acid Composition

Fatty acid content was determined as described by Liu et al. [36]. Briefly, cells from 20 mL culture were harvested on day 7, freeze-dried, and homogenized. Total lipids were extracted with chloroform–methanol (2:1, *v*/*v*), and the dried extract was transesterified with sulfuric acid–methanol (5:95, *v*/*v*) at 70 °C for 4 h. Fatty acid methyl esters were extracted with hexane and analyzed by GC-MS (Agilent 5977C, Santa Clara, CA, USA) on a DB-FATWAX UI column (30 m × 0.25 mm × 0.25 µm) in selected ion monitoring (SIM) mode. The oven temperature program was as follows: initial hold at 50 °C for 3 min, ramped at 25 °C/min to 180 °C and held for 3 min, then ramped at 2 °C/min to 230 °C and held for 7 min. The inlet temperature was 260 °C, and the MSD transfer line temperature was 240 °C. Helium was used as the carrier gas at a flow rate of 1 mL/min with a split ratio of 20:1. Identification and quantification were performed by comparing retention times and mass spectra with a commercial FAME standard (CRM47885, MilliporeSigma, Burlington, MA, USA) and the NIST mass spectral database (National Institute of Standards and Technology, Gaithersburg, MD, USA).

### 3.13. Metabolic Profiling

Metabolite extraction and derivatization were performed based on the method of Lv et al. [81] with minor modifications. Algal cells were collected from 10 mL of culture by centrifugation on days 3, 5 and 7 post high-light exposure. The resulting pellet was freeze-dried, and the dried biomass was homogenized in a 2 mL centrifuge tube using a bead mill. Metabolites were extracted with 1 mL of methanol containing ribitol (0.012 mg/mL) as an internal standard. After sequential vortexing, ultrasonication, and shaking, the homogenate was centrifuged at 11,000× *g* for 10 min. A 500 μL aliquot of supernatant was mixed with 375 μL of chloroform and 750 μL of distilled water. Phase separation was achieved by centrifugation, and 150 μL of the upper polar phase was collected, passed through a 0.22 μm membrane filter, and dried under vacuum. Derivatization was performed in two steps: first, the sample was incubated with 40 μL of methoxyamine hydrochloride (20 mg/mL in pyridine, Sigma-Aldrich, St. Louis, MO, USA) at 37 °C for 2 h; subsequently, it was derivatized with 70 μL of N-methyl-N-(trimethylsilyl)trifluoroacetamide (MSTFA, Sigma-Aldrich, St. Louis, MO, USA) at 37 °C for 30 min.

GC-MS analysis was carried out using an Agilent 5977C system equipped with a HP-5MS UI capillary column (30 m × 0.25 mm × 0.25 μm). High-purity helium was used as the carrier gas at a constant flow rate of 2 mL/min. The oven temperature was programmed as follows: initial hold at 80 °C for 2 min, ramped to 330 °C at a rate of 15 °C/min, and finally held at 330 °C for 6 min. Data were acquired in full scan mode. Metabolites were identified by comparison mass spectra and retention indices with those from commercial standards, the NIST Mass Spectral Database (National Institute of Standards and Technology, Gaithersburg, MD, USA), and the Golm Metabolome Database (http://gmd.mpimp-golm.mpg.de/, accessed on 10 August 2025). Quantitative ions and retention times for all identified metabolites are provided in Supplementary Table S2. Peak areas were normalized to the internal standard ribitol (Sigma-Aldrich, St. Louis, MO, USA) for relative quantification.

### 3.14. Comparative Transcriptomic Analysis

Transcriptomic analysis was performed on algal cells harvested after 48 h of high-light exposure. RNA extraction, sequencing, and data processing followed previously described methods [23]. Gene expression levels were quantified as fragments per kilobase of transcript per million mapped reads (FPKM) values. Differentially expressed genes (DEGs) were identified using thresholds of |log_2_(fold change)| ≥ 1 and Q-value ≤ 0.001. Gene Ontology (GO) and KEGG pathway enrichment analyses were conducted with a significance cutoff of Q-value ≤ 0.05, using KOBAS 2.0 for multiple testing correction. Raw transcriptomic data generated in this study are provided in the Supplementary Materials (Raw_Transcriptomic_Data).

### 3.15. Statistical Analysis

Unless otherwise specified, all experiments included three independent biological replicates. Data are presented as mean ± standard deviation. Statistical analyses were performed with SPSS 27 software (IBM Corp., Armonk, NY, USA), and comparisons between two groups at the same time points were evaluated with Student’s *t*-test. Significance levels were defined as *, *p* < 0.05 and **, *p* < 0.01. For multivariate analysis, both the GC-MS-derived peak areas of all identified metabolites and the FPKM values of all genes from RNA-seq were separately imported into SIMCA-P software (version 14.1, Umetrics, Umeå, Sweden). Prior to PCA, the data were mean-centered and scaled to unit variance (UV scaling) using the software’s default setting. PCA score plots were generated with Hotelling’s T^2^ ellipses (95% confidence interval) to visualize group separation. The variance explained by each principal component is indicated in the score plots. Graphs were generated using Origin 2026 (OriginLab Corporation, Northampton, MA, USA) and edited by Adobe Illustrator 2020 software (Adobe, San Jose, CA, USA).

## 4. Conclusions

This study demonstrates that the combined application of NaCl and CMP effectively reprograms central metabolism and gene expression in *H. lacustris* under continuous high-light stress. The optimized combination (0.5 g/L NaCl and 0.5 mM CMP), identified via an orthogonal design, synergistically enhanced the co-production of astaxanthin and total fatty acids while significantly suppressing SCW formation. Relative to the CK, the NC treatment increased astaxanthin yield by more than 35.6% and total fatty acid yield by 28%, while maintaining 96.8% of cells in a motile state without SCW. These effects were accompanied by elevated ROS levels, concomitant with higher Fv’/Fm’ and rETR(II), along with enhanced NPQ capacity, and drove a reprogramming of carbon metabolism. Metabolomic profiling indicated reduced carbon supply for SCW polysaccharide biosynthesis, coupled with decreased protein yield and altered amino acid profiles characteristic of nitrogen-limited metabolism, which is consistent with a potential reallocation of carbon resources toward high-value products (fatty acids and astaxanthin). Transcriptomic analysis further revealed the downregulation of key genes involved in biosynthetic pathways of SCW components (including mannan and algaenan) and the significant upregulation of genes in the MEP and astaxanthin biosynthetic pathways. Scale-up experiments validated the feasibility of cultivating astaxanthin-rich motile cells using this strategy in a photobioreactor. In summary, the synergistic application of NaCl and CMP presents an efficient upstream metabolic regulation strategy for the concurrent enhancement of astaxanthin and fatty acid production in *H. lacustris*, providing a promising approach for sustainable microalgal biorefinery.

## Figures and Tables

**Table 1:** Effects of different NaCl and CMP concentration combinations on astaxanthin yield, motile cell proportion, and desirability, along with two-way ANOVA results, on day 7 post high-light exposure.

NaCl Concentration (g/L)	CMP Concentration (mM)	Astaxanthin Yield (mg/L)	Motile Cell Proportion (%)	Desirability
0	0	11.14 ± 0.87	3.08 ± 0.84	0.11
	0.25	10.08 ± 0.77	76.68 ± 1.12 **	0.53
	0.50	10.11 ± 0.34	89.45 ± 0.97 **	0.58
	1.00	10.07 ± 0.50	96.75 ± 1.48 **	0.60
0.25	0	12.37 ± 0.60	1.83 ± 1.13	0.08
	0.25	9.48 ± 0.81	73.11 ± 2.33 **	0.47
	0.50	8.97 ± 0.28 *	86.83 ± 1.51 **	0.46
	1.00	7.00 ± 0.35 **	92.70 ± 0.77 **	0.19
0.50	0	15.40 ± 0.68 **	5.08 ± 0.87	0.20
	0.25	14.91 ± 0.52 **	70.72 ± 1.34 **	0.79
	0.50	15.11 ± 0.43 **	96.83 ± 1.53 **	0.94
	1.00	13.52 ± 0.43 *	91.23 ± 0.56 **	0.82
1.00	0	14.94 ± 0.67 **	4.22 ± 0.27	0.18
	0.25	9.96 ± 0.61	69.05 ± 1.74 **	0.50
	0.50	9.56 ± 0.90	89.22 ± 1.71 **	0.53
	1.00	7.25 ± 0.74 **	80.23 ± 1.82 **	0.23
ANOVA	NaCl	*p* < 0.0001	*p* < 0.0001	
	CMP	*p* < 0.0001	*p* < 0.0001	
	NaCl × CMP	*p* < 0.0001	*p* < 0.0001	

CMP, cytidine monophosphate. * *p* < 0.05, ** *p* < 0.01, vs. CK group.

**Table 2:** Statistical comparison of fatty acid levels between NC and CK groups on day 7 post high-light exposure.

Fatty Acids	Abbreviation	CK (mg/L)	NC (mg/L)	Ratio (NC/CK)	*p*-Value
Myristic acid	C14:0	1.489 ± 0.055	2.225 ± 0.038	1.49	<0.001
Pentadecanoic acid	C15:0	0.416 ± 0.014	0.494 ± 0.012	1.19	<0.001
Palmitic acid	C16:0	53.102 ± 2.064	70.824 ± 1.644	1.33	<0.001
Palmitoleic acid	C16:1 (isomer 1)	0.336 ± 0.048	0.477 ± 0.029	1.42	0.002
7-Hexadecenoic acid	C16:1 (isomer 2)	0.156 ± 0.012	0.185 ± 0.012	1.18	0.015
Hexadecadienoic acid	C16:2	0.806 ± 0.067	0.581 ± 0.038	0.72	0.001
4,7,10-Hexadecatrienoic acid	C16:3 (isomer 1)	0.958 ± 0.070	0.904 ± 0.034	0.94	0.244
Roughanic acid	C16:3 (isomer 2)	1.475 ± 0.101	1.724 ± 0.043	1.17	0.003
Hexadecatetraenoic acid	C16:4	12.311 ± 0.783	13.851 ± 0.499	1.13	0.012
Stearic acid	C18:0	8.695 ± 0.596	9.858 ± 0.283	1.13	0.014
Oleic acid	C18:1 (isomer 1)	4.550 ± 0.247	8.252 ± 0.166	1.81	<0.001
Vaccenic acid	C18:1 (isomer 2)	2.148 ± 0.186	3.536 ± 0.098	1.65	<0.001
Linoleic acid	C18:2	19.902 ± 1.200	24.143 ± 0.511	1.21	<0.001
γ-Linolenic acid	C18:3 (isomer 1)	1.045 ± 0.094	2.034 ± 0.066	1.95	<0.001
Linolenic acid	C18:3 (isomer 2)	21.388 ± 1.287	25.685 ± 0.810	1.20	0.001
Octadecatetraenoic acid	C18:4	3.151 ± 0.233	4.416 ± 0.128	1.40	<0.001
Arachidic acid	C20:0	0.466 ± 0.013	0.452 ± 0.014	0.97	0.417
Dihomo-gamma-linolenic acid	C20:3	0.173 ± 0.017	0.092 ± 0.004	0.53	<0.001
Arachidonic acid	C20:4	0.742 ± 0.063	0.830 ± 0.036	1.12	0.056
Eicosapentaenoic acid	C20:5	0.784 ± 0.069	0.758 ± 0.037	0.97	0.667
Tetracosanoic acid	C24:0	0.154 ± 0.012	0.230 ± 0.010	1.49	<0.001
Monounsaturated fatty acids	MUFAs	7.191 ± 0.423	12.449 ± 0.207	1.73	<0.001
Polyunsaturated fatty acids	PUFAs	62.736 ± 3.964	75.019 ± 2.057	1.20	0.001
Unsaturated fatty acids	UFAs	69.927 ± 4.363	87.469 ± 2.228	1.25	<0.001
Saturated fatty acids	SFAs	64.322 ± 2.599	84.083 ± 1.865	1.31	<0.001
Total fatty acids	TFAs	134.249 ± 6.829	171.552 ± 3.996	1.28	<0.001

Red and green cells denote the metabolites were significantly higher and lower in the NC group compared to those in the CK group, respectively.

**Table 3:** Statistical comparison of metabolite levels between NC and CK groups.

Classes	Metabolites	Ratio (NC/CK)			Two-Way ANOVA		
		Day 3	Day 5	Day 7	Treat	Time	Interaction
Sugars	Glucose	0.6	0.64	0.73	**	**	n.s.
	Fructose	0.12	0.52	0.68	**	**	*
	Sucrose	0.15	0.68	1.03	**	**	**
	Mannose	0.12	0.59	0.77	**	**	**
Amino acids and their derivatives	Hydroxyproline	1.11	1.12	0.9	n.s.	*	**
	Methionine	1.08	1.04	0.9	n.s.	*	**
	Lysine	1.32	1.07	0.53	**	**	**
	Cysteine	0.33	1.98	1.39	*	**	**
	Serine	0.32	2.02	1.34	**	**	**
	beta-Alanine	0.79	2.46	2.4	**	**	**
	Alanine	0.65	1.38	1.15	n.s.	**	**
	Valine	1.05	0.98	0.51	**	n.s.	**
	Leucine	0.89	0.77	0.65	**	n.s.	*
	Isoleucine	0.98	0.8	0.53	**	**	**
	Glycine	0.75	1.1	0.9	*	**	**
	Tryptophan	0.17	0.68	1.02	**	**	**
	Tyrosine	0.92	1.09	0.74	n.s.	n.s.	**
	Pyroglutamic acid	0.77	0.69	0.65	**	**	**
TCA-cycle and related organic acids	Pyruvic acid	0.98	1.02	0.99	n.s.	n.s.	n.s.
	Succinic acid	0.54	2.21	1.32	n.s.	*	n.s.
	Malic acid	0.59	0.69	0.85	**	**	*
	Malonic acid	1.03	0.86	0.78	**	**	**
	Glyceric acid	1.13	0.51	0.51	**	*	*
	Threonic acid	0.94	0.21	0.41	**	**	**
	Citric acid	0.19	0.59	0.68	**	**	**
Others	Sorbitol	1.01	0.65	0.69	*	**	**
	Threitol	0.35	0.6	0.68	**	**	**
	Phosphoric acid	1.57	1.01	1.06	**	**	**
	Glycerol	0.33	2.03	1.33	*	**	**
	Urea	1.07	1.05	0.87	**	**	n.s.

Red and green cells denote metabolites that were significantly higher and lower in the NC group compared to those in the CK group, respectively. Metabolites for which no significant difference was observed between the NC and CK groups at any of the three time points are not listed in the table. * *p* < 0.05; ** *p* < 0.01; n.s., not significant.

## Data Availability

The original contributions presented in this study are included in the article/Supplementary Materials.

## Supplementary Materials

The following supporting information can be downloaded at: https://zenodo.org/records/22013384 (accessed on 20 July 2026). The “Supplementary figures” file contains the following six figures: Figure S1. Representative light microscopy images of *H. lacustris* cells on day 0 prior to high-light induction; Figure S2. Appearance of *H. lacustris* cultures in the 3-L photobioreactor on day 7; Figure S3. Representative light microscopy images of *H. lacustris* cells sampled from the 3-L photobioreactor cultures on day 7; Figure S4. Dry weight (A) and cell density (B) of NC and CK groups at different time points; Figure S5. Effects of NaCl and CMP combination on the expression of genes involved in purine metabolism; Figure S6. Effects of NaCl and CMP combination on the expression of genes involved in pyrimidine metabolism. The “Supplementary tables” file contains the following seven tables: Table S1. Statistical comparison of fatty acid levels between NC and CK groups; Table S2. Characterization of metabolites identified by GC-MS analysis; Table S3. GO terms enriched by upregulated genes; Table S4. GO terms enriched by downregulated genes; Table S5. KEGG pathway enriched by downregulated genes; Table S6. Differentially expressed genes in purine metabolism; Table S7. Differentially expressed genes in pyrimidine metabolism. The “Raw_transcriptomic_data” file contains the raw RNA-seq data generated in this study.

## Abbreviations

The following abbreviations are used in this manuscript:

ACACA	Acetyl-CoA carboxylase
ACSL	Long-chain acyl-CoA synthetase
ANOVA	Analysis of variance
BHY	Beta-carotene hydroxylase
BKT	Beta-carotene ketolase
CK	The untreated control group
CMP	Cytidine monophosphate
CRTISO	Prolycopene isomerase
DEGs	Differentially expressed genes
DMSO	Dimethyl sulfoxide
DRGs	Down-regulated genes
DW	Dry weight
ECM	Extracellular matrix
FA	Fatty acid
FASN	Fatty acid synthase
FDPS	Farnesyl diphosphate synthase
FPKM	Fragments per kilobase of transcript per million mapped reads
Fv/Fm	Maximum photochemical efficiency
Fv′/Fm′	Effective photochemical efficiency
GC-MS	Gas Chromatography–Mass Spectrometry
GDP-mannose	Guanosine diphosphate mannose
GGPP	Geranylgeranyl pyrophosphate
GMPP	Mannose-1-phosphate guanylyltransferase
GO	Gene Ontology
H2DCFDA	2′,7′-dichlorodihydrofluorescein diacetate
HACD	3-hydroxyacyl-CoA dehydrogenase
ISPD	2-C-methyl-D-erythritol 4-phosphate cytidylyltransferase
ISPF	2-C-methyl-D-erythritol 2,4-cyclodiphosphate synthase
ISPH	4-hydroxy-3-methylbut-2-enyl diphosphate reductase
KCS	3-ketoacyl-CoA synthase
KEGG	Kyoto Encyclopedia of Genes and Genomes
LCYB	Lycopene beta-cyclase
LED	Light-emitting diode
MEP	Methylerythritol 4-phosphate
MNN1	Alpha-1,3-mannosyltransferase
MNN2	Alpha-1,2-mannosyltransferase
MPI	Mannose-6-phosphate isomerase
MUFAs	Monounsaturated fatty acids
NaCl	Sodium chloride
NC	The combination of 0.5 g/L NaCl and 0.5 mM CMP group
NPQ	Non-photochemical quenching
OCH1	Alpha-1,6-mannosyltransferase
PC	Principal component
PCA	Principal component analysis
GPI	Glucose-6-phosphate isomerase
PUFAs	Polyunsaturated fatty acids
rETR	Relative electron transport rate
ROS	Reactive oxygen species
SACA	Sucrose-6-phosphate hydrolase
SCW	Secondary cell wall
scrK	Fructokinase
SFAs	Saturated fatty acids
SPP	Sucrose phosphatase
SPS	Sucrose-phosphate synthase
TCA	Tricarboxylic acid
UFAs	Unsaturated fatty acids
URGs	Up-regulated genes
VVM	Volume of air per volume of medium per minute
ZEP	Zeaxanthin epoxidase
